# Fate of Barium Sulfate Nanoparticles Deposited in the Lungs of Rats

**DOI:** 10.1038/s41598-019-44551-2

**Published:** 2019-06-03

**Authors:** Ramon M. Molina, Nagarjun V. Konduru, Priscila M. Queiroz, Benjamin Figueroa, Dan Fu, Lan Ma-Hock, Sibylle Groeters, Dirk Schaudien, Joseph D. Brain

**Affiliations:** 1 Molecular and Integrative Physiological Sciences Program, Department of Environmental Health, Harvard T.H. Chan School of Public Health, 665 Huntington Avenue, Boston, MA 02115 USA; 2 Department of Chemistry, University of Washington, 36 Bagley Hall, Seattle, WA 98195 USA; 3 BASF SE, Carl-Bosch-Straße 38, 67056 Ludwigshafen, Germany; 4 Fraunhofer-Institute for Toxicology and Experimental Medicine ITEM Nikolai-Fuchs-Str. 1, 30625 Hannover, Germany

**Keywords:** Respiration, Bone

## Abstract

We have shown that barium [from BaSO_4_ nanoparticles (NPs)] was cleared from the lungs faster than other poorly soluble NPs and translocated mostly to bone. We now studied barium biokinetics in rats during Study 1: two-year inhalation exposure to 50 mg/m^3^ BaSO_4_ NP aerosols, and Study 2: single intratracheal (IT) instillation of increasing doses of BaSO_4_ NPs or BaCl_2_. Study 1 showed that lung barium content measured by inductively coupled plasma mass spectrometry increased during 360 days of BaSO_4_ NP aerosol exposures. An equilibrium was established from that time until 2 years. Barium concentrations in BaSO_4_-exposed animals were in the order (lungs > lymph nodes > hard bone > bone marrow > liver). In Study 2, there was an increase in lung barium post-IT instillation of BaSO_4_ NPs while barium from BaCl_2_ was mostly cleared by day 28. Transmission electron microscopy showed intact BaSO_4_ NPs in alveolar macrophages and type II epithelial cells, and in tracheobronchial lymph nodes. Using stimulated Raman scattering microscopy, specific BaSO_4_ Raman spectra were detected in BaSO_4_ NP-instilled lungs and not in other organs. Thus, we posit that barium from BaSO_4_ NPs translocates from the lungs mainly after dissolution. Barium ions are then incorporated mostly into the bone and other organs.

## Introduction

Barium sulfate particles are usually included in the family of poorly soluble particles (PSP) or poorly soluble low toxicity (PSLT) particles. These categories also include cerium dioxide (CeO_2_) and titanium dioxide (TiO_2_)^1–3^. These so-called biodurable nanomaterials are thought to be poorly absorbed into the blood from the gut or lungs^4–6^. Determining the fate of BaSO_4_ NPs is important because they have extensive commercial applications. Barium sulfate nanoparticles (BaSO_4_ NPs) are used as fillers in coatings (e.g., in motor vehicles), in orthopedic medicine, diagnostic imaging and other applications^7–10^. It has been reported that pellethane, a polyurethane elastomer, when incorporated with BaSO_4_ NPs exhibits antimicrobial properties *in vitro*^11^.

The toxicity and biokinetics of nanoparticles are influenced by their physicochemical properties^12–16^. Poorly soluble particles usually have slower clearance kinetics and diminished biological effects compared to soluble particles^3,17,18^. For example, CeO_2_ and far more soluble ZnO NPs of similar size were cleared at vastly different rates from the lungs. Rapidly dissolving ZnO NPs elicited more acute pulmonary inflammation, and disappeared much faster that CeO_2_ NPs^19,20^. We have shown that BaSO_4_ NPs instilled into the lungs of rats elicited less injury and inflammation than do CeO_2_ NPs of similar primary NP size and amount^18,20^. Finally, particles of different sizes with the same composition elicit different biological responses^21–23^. Dissolution rates and biologic responses increase as particle size decreases at the same mass dose. Moreover, the lung clearance of radioactive barium after intratracheal (IT) instillation of ^131^BaSO_4_ was influenced by particle size^24,25^. These two studies produced retention half-lives of radioactive 1.45 µm and 3.6 µm ^133^BaSO_4_ particles of approximately 2 and 10 days, respectively. Higher initial lung burden of the larger particles also increased the half-life from 10 to 27 days^25^.

To explore the relative toxicity of BaSO_4_ (NM-220) and CeO_2_ (NM-212) NPs for manufacturers as well as consumers, a long-term project was funded by the German Federal Ministry of Education and Research. This project is managed within the Organization for Economic Cooperation and Development (OECD) and the European Union Project NANoREG (a Europe-wide approach to the regulatory testing of manufactured nanomaterials). An inhalation study in rats and associated analytic procedures were conducted at BASF SE, Experimental Toxicology and Ecology Ludwigshafen, Germany. The NM-220 and NM-212 used in this project are well characterized nanomaterials from the European Commission Joint Research Center (JRC) nanomaterial (NM) repository (Ispra, Italy). Both CeO_2_ (NM-212) and BaSO_4_ (NM-220) have low water solubility^18,20^. In the earlier phase of this project, we found that despite the reported low solubility of BaSO_4_ NPs, barium was cleared from the lungs much more rapidly compared to other poorly soluble nanoparticles, including CeO_2_^3,18,20^. Since it was established in short-term inhalation studies that BaSO_4_ does not cause adverse health effects^26–28^ at the doses studied, a concentration of 50 mg/m^3^ was selected by BASF SE for the 2-year repeated inhalation exposures, a level at which lung overload might be expected.

We recently showed that lung clearance of ^131^Ba post-instillation of ^131^BaSO_4_ NPs was significantly faster than of ^141^Ce post-instillation of ^141^CeO_2_ NPs at the same mass dose in the same male rat strain and age^18,20^. Interestingly, we found that the major sites of retention of barium translocated from the lungs were bone, bone marrow, and tracheobronchial and mediastinal lymph nodes. These biokinetic data were based on tissue ^131^Ba (radioactivity) or Ba measurements by inductively coupled plasma-mass spectrometry (ICP-MS). Neither method could differentiate whether the measured barium was present in intact BaSO_4_ NPs, ionic barium or new structural forms containing Ba.

The aims of our research are: Study 1 to measure retention of barium in multiple organs of rats that inhaled BaSO_4_ NPs during the 2-year inhalation exposure described earlier and Study 2 to examine the role of BaSO_4_ particle dissolution in the translocation of barium from the lungs to extra-pulmonary tissues especially the bone and lymph nodes. A common goal in both studies is to characterize the chemical form and morphology of retained barium in the lungs, bone, and tracheobronchial lymph nodes. To pursue the first aim, we measured barium concentrations in the lungs and selected organs in rats from the 2-year inhalation study at BASF SE. In the second aim, we compared lung and bone concentrations of barium in rats IT-instilled with BaSO_4_ NPs versus BaCl_2_ solution. Finally, we used electron microscopy and stimulated Raman Scattering (SRS) microscopy to provide qualitative insights into the chemical form of barium in the lungs and other tissues. Our overall goal was to explore the mechanisms of clearance of BaSO_4_ NPs from the lungs and to quantify and understand the mechanisms for the retention of barium in extrapulmonary organs.

## Results

### Study 1: Long-term inhalation exposure to BaSO_4_ aerosols

#### Biokinetics of inhaled BaSO_4_ NPs

A complete physicochemical characterizations of BaSO_4_ NPs (NM-220) has been reported^18,28^. Selected physicochemical endpoints for NM-220 and the similar reproduced BaSO_4_ NPs used in Study 1 are summarized in Table S1 (Supplement). For the long-term inhalation study, the particle concentrations and size distribution of generated BaSO_4_ aerosols are summarized in Table 1. The target concentration of about 50 mg/m^3^ was maintained throughout the inhalation exposures. The particle size distribution of aerosolized BaSO_4_ NP aggregates had an approximate MMAD of 2.0 µm and thus was in the respirable range for rats.

**Table 1:** Aerosol concentrations and particle size distributions of BaSO_4_ NM-220.

Duration of Exposure	Targeted concentrations (mg/m³)	Measured concentrations (mg/m³)	MMAD/GSD mean (µm)	Particle count concentration (particle/cm³)	Particle count median diameter (nm)
2 years	50	50.3 ± 5.8	2.0/2.0	45,900	341

Based on 16 measurements (10 replicates) over the two-year period. Data are mean ± SD.

**Figure 1:**
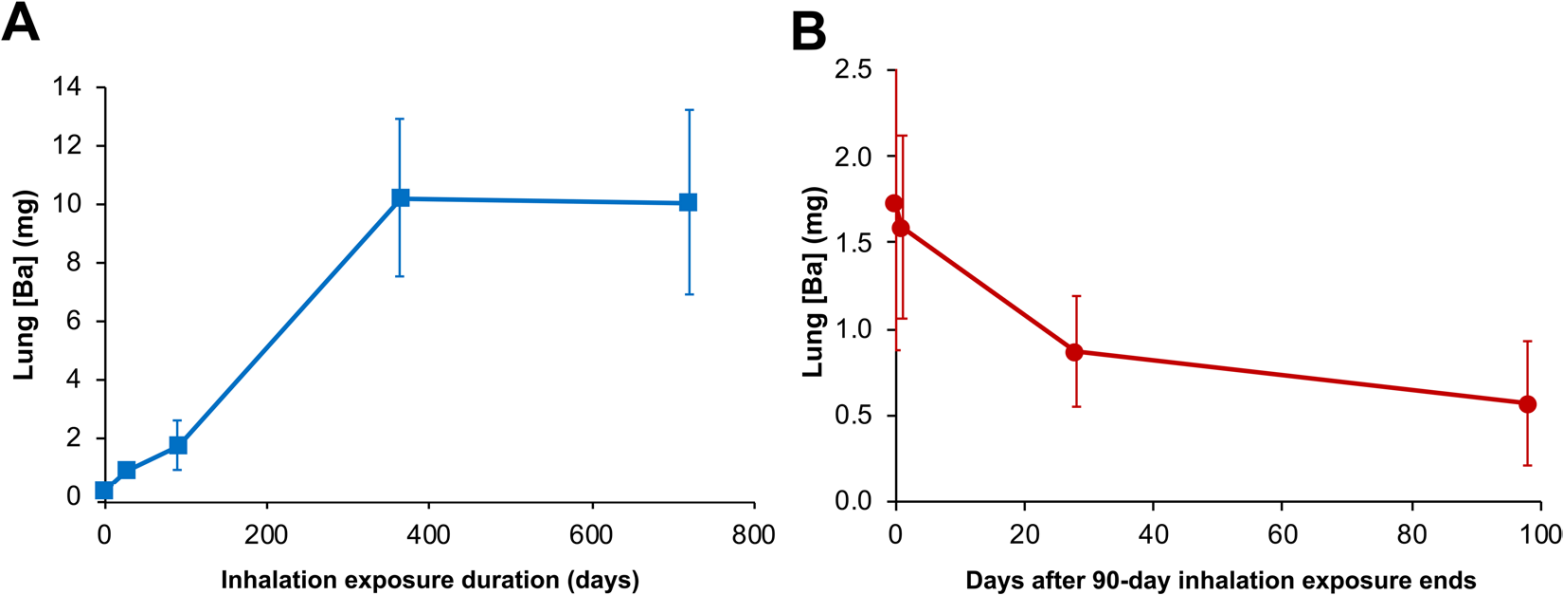
Study 1. (**A**) Cumulative retention of barium in the lungs of rats during inhalation exposures to 50 mg/m^3^ BaSO_4_ NPs aerosols or filtered air. Lung burdens of barium in BaSO_4_ NPs aerosol exposed rats examined at 1, 28, 90, 364 and 730 days and air-exposed rats at 730 days. Air-exposed rat lungs had only 0.04 ± 0.01 µg barium. (**B**) Clearance of retained barium after 90-day multiple inhalation exposures to 50 mg/m^3^ barium sulfate nanoparticles. The retained barium at 90 days was 1.6 mg/lung (1.24 mg/g lung). The clearance half-time after 90 days exposure was approximately 56 days. This clearance graph was based on data from reference^28^.

After the final day of the multiple inhalation exposure of rats for 2 years, four BaSO_4_ aerosol-exposed and two filtered air-exposed controls were euthanized, and samples were sent to Harvard for further analyses. The barium concentrations and estimated total barium in the lungs, liver, lymph nodes, bone and bone marrow were measured. Based on these current data obtained at 2 years and on the previous reports in rats examined at 1, 28, and 90 days^18,28^, we constructed a cumulative lung retention curve over the two-year period (Fig. 1A). The combined data showed that barium accumulated in the lungs with time up to 360 days. An equilibrium was established from that point until 730 days. During that period, deposition rate equaled clearance rate. Tissue concentrations and total amounts of barium in the lungs, bone (without marrow), bone marrow, liver and lymph nodes are shown in Table 2. Detectable amounts of barium in these organs were also present in air-exposed control rats almost certainly from background air borne and dietary sources. In both air-exposed controls and BaSO_4_-exposed rats, barium concentrations significantly differed among the organs we examined (ANOVA, P = 0.04). Additionally, significantly greater barium was found in the lungs (P = 0.003) and bones (P = 0.038) of BaSO_4_-exposed than in air-exposed controls. The highest barium concentrations in the BaSO_4_-exposed animals were measured in the lungs and lymph nodes (tracheobronchial and mediastinal), followed by the hard bone and bone marrow, while the liver had the lowest concentration. The total lung and liver barium contents were based on actual organ weights while those for bone and bone marrow were computed using estimates of their tissue percentage of total body weight: 3.2% bone marrow and 5% bone^29^. The highest amounts of barium from the exposed animals were found in the lungs as expected, and in hard bone. Based on reported lung barium data from the same 2-year inhalation study (Study 1)^28^, we generated a lung clearance curve for barium after multiple inhalation exposures over 90 days (Fig. 1B). The total lung barium and concentration at day 90 were 1.60 mg/lung and 1.24 mg/g, respectively. The clearance half-time from that time point onward was approximately 56 days. Since rats were unavailable after the end of the inhalation exposure, lung clearance when retained barium is higher could not be determined.

**Table 2:** Barium concentration and content in the lungs and selected organs of rats after chronic exposures to 50 mg/m^3^ BaSO_4_ NPs or filtered air (6 h/day, 5 consecutive days/week) for 2 years.

Study 1	Filtered Air			BaSO_4_		
	[Ba] µg/g	Organ Wt. (g)	Total Ba (µg)	[Ba] µg/g	Organ Wt. (g)	Total Ba (µg)
Lungs	0.04 ± 0.01^#^	1.11 ± 0.12	0.04 ± 0.01^#^	5808 ± 2552^#,^*	1.91 ± 0.78	10056 ± 3148^#^
Bone	7.33 ± 0.62	18.35 ± 0.29	134.6 ± 13.4	348 ± 70.1*	15.90 ± 1.34	5546 ± 1348
Bone Marrow	6.24 ± 4.58	11.74 ± 0.19	72.8 ± 52.7	73.2 ± 109	10.18 ± 0.86	781 ± 1199
Liver	0.04 ± 0.03	11.22 ± 0.22	0.40 ± 0.31	0.12 ± 0.05	9.41 ± 0.63	1.06 ± 0.39
Lymph Nodes	7.48 ± 1.82	N.D.	N.D.	4461 ± 3492	N.D.	N.D.

Data are mean ± SD, n = 2 filtered air-exposed control, n = 4 BaSO_4_ NP-exposed rats.

Barium concentration in tracheobronchial and mesenteric lymph nodes were pooled.

Bone and bone marrow weights were estimated as 5% and 3.2% of body weight, respectively.

N.D., not determined, estimate of total lymph node mass in rats is not available.

*P < 0.05, Student’s T test, air-exposed vs. BaSO_4_-exposed.

^#^P < 0.05, ANOVA, significant differences among organs.

#### Transmission electron microscopic examination of tissues from rats in Study 1

**Figure 2:**
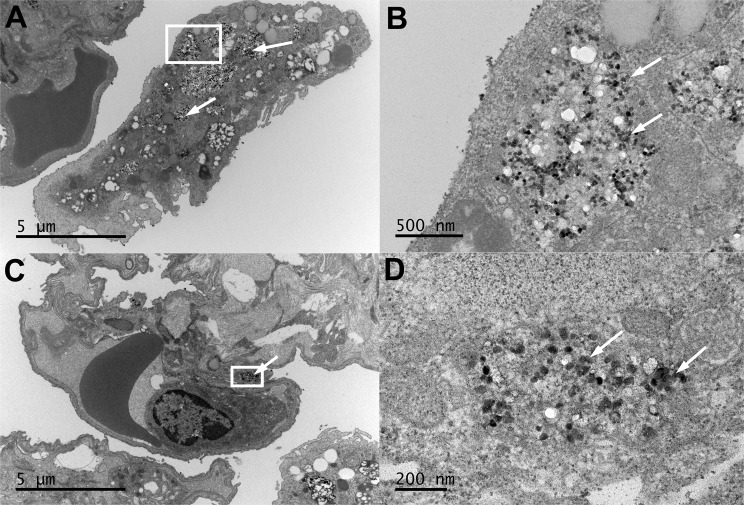
Study 1. Rat lung after 24 months of multiple inhalation exposures to 50 mg/m^3^ BaSO_4_ NPs aerosols. (**A**) An alveolar macrophage with engulfed BaSO_4_ nanoparticles (arrows) within phagolysosomes. (**B**) Higher magnification of boxed area in panel A showing BaSO_4_ particles (arrows). (**C**) Type II epithelial cell with internalized BaSO_4_ nanoparticles (arrows). (**D**) Higher magnification of boxed area in panel C showing BaSO_4_ particles (arrows) within a type 2 epithelial cell.

**Figure 3:**
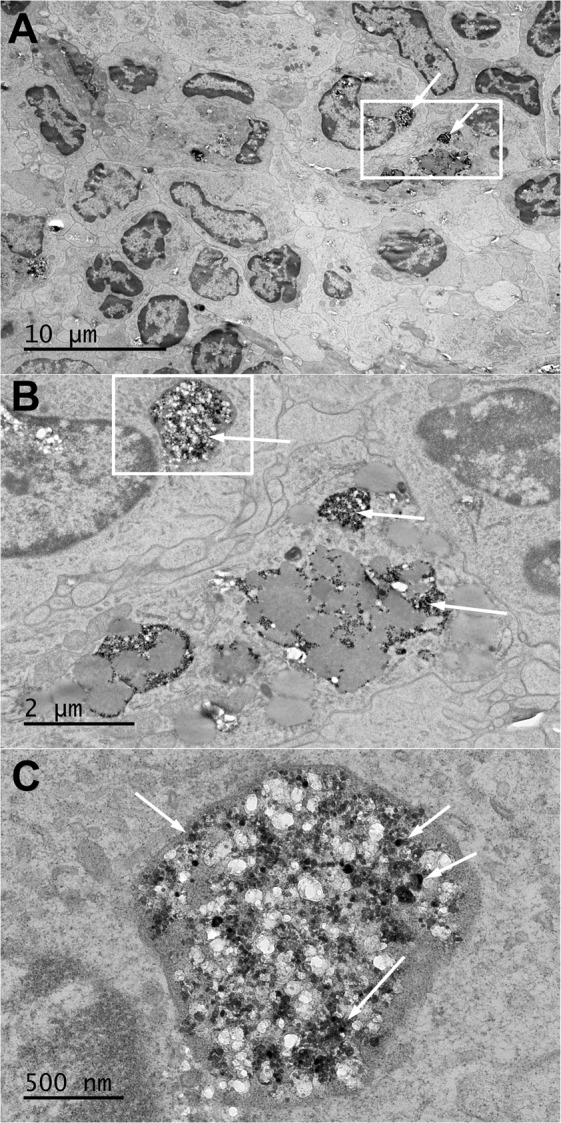
Study 1. Rat tracheobronchial lymph node after 24 months of multiple inhalation exposures to 50 mg/m^3^ BaSO_4_ NPs aerosols. (**A**) A section of lymph node showing phagocytic cells with engulfed BaSO_4_ nanoparticles (arrows) within phagolysosomes. (**B**) Higher magnification of boxed area in panel A showing abundant BaSO_4_ particles in membrane-bound structures (arrows). (**C**) Higher magnification of boxed area in panel B showing BaSO_4_ particles (arrows) within a phagocytic cell cytoplasm.

The distribution of retained BaSO_4_ NPs in the lungs of rats was examined by transmission electron microscopy. Figure 2 shows a rat lung one day after the final inhalation exposure to 50 mg/m^3^ BaSO_4_ NPs aerosols that lasted for two years. The majority of the particles were seen within phagolysosomes in alveolar macrophages (Fig. 2A,B). BaSO_4_ NPs were occasionally observed in type II epithelial cells (Fig. 2C,D). The majority of observed BaSO_4_ NPs were seen within phagolysosomes in macrophages and occasionally in neutrophils (data not shown). Since barium concentrations in both tracheobronchial and mesenteric lymph nodes were nearly as high as in the lungs, ultrastructural examination with TEM was also performed. We observed BaSO_4_ NPs within endosomes of phagocytic cells in tracheobronchial lymph nodes (Fig. 3). No evidence of particles was seen in the mesenteric lymph nodes despite the presence of a high concentration of barium measured by ICP-MS. No evidence of particles was seen in the bone marrow, the liver or the lungs of air-exposed controls.

### Study 2: Intratracheal instillation of BaSO_4_ nanoparticles versus soluble BaCl_2_ in rats

#### Biokinetics of IT- instilled BaSO_4_ nanoparticles versus soluble BaCl_2_

**Figure 4:**
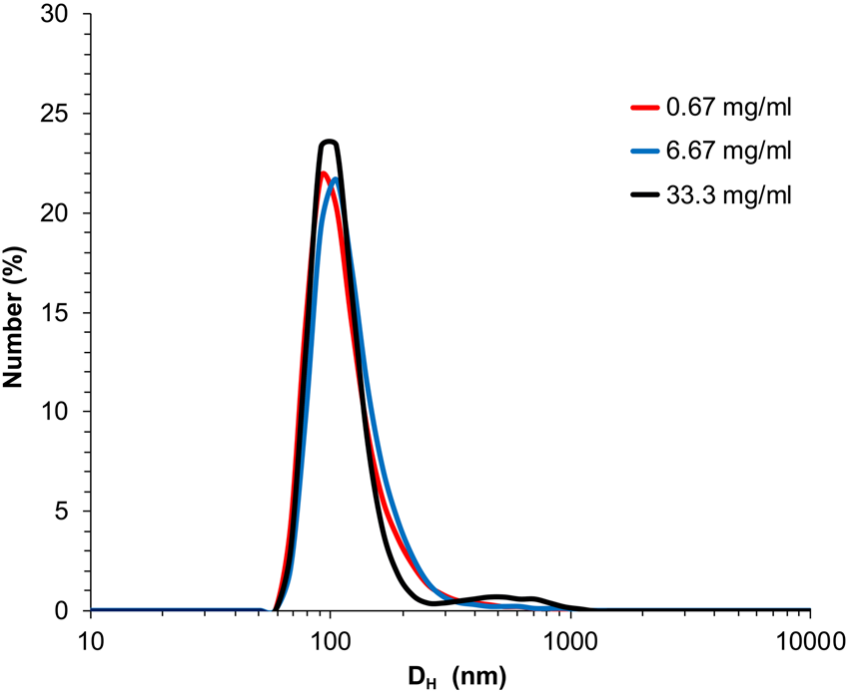
Study 2. Dynamic light scattering size distribution analyses of BaSO_4_ nanoparticle suspensions in distilled water after sonication at 242 J/ml. Besides hydrodynamic diameters shown here, zeta potentials, polydispersity index and conductance were also measured (See Table 3).

**Figure 5:**
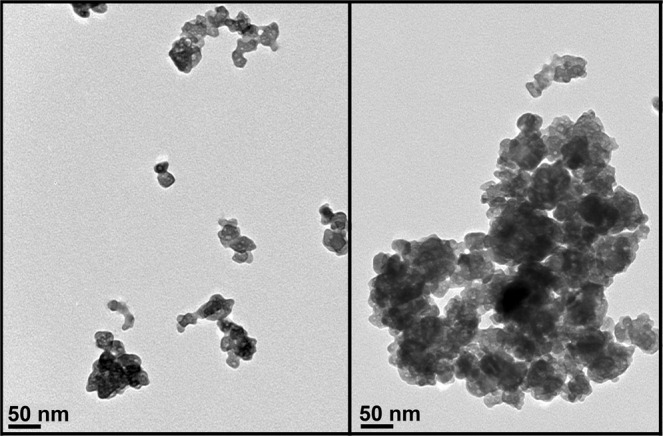
Study 2. TEM micrograph of sonicated suspension of 0.67 mg/ml BaSO_4_ NPs in distilled water. Left panel shows individual particles (~25 nm) and small agglomerates. The right panel shows a bigger agglomerate of BaSO_4_ nanoparticles seen in a more concentrated 33.3 mg/ml suspension.

Characterization of BaSO_4_ NP (NM-220 batch) suspensions used in Study 2 is summarized in Table 3. The agglomerate sizes were assessed using dynamic light scattering (DLS). We found that the agglomerate sizes were influenced by particle concentration: the higher the concentration, the larger the hydrodynamic diameter (Table 3, Fig. 4). The most concentrated 33.3 mg/ml suspension showed a bimodal distribution indicating the presence of two distinct populations of different sized particle agglomerates. Transmission electron micrographs of BaSO_4_ NPs suspended in distilled water show a non-spherical globular shape of individual nanoparticles (Fig. 5). They appeared as individual particles or as agglomerates of variable sizes that roughly corresponded to the range of hydrodynamic sizes obtained by DLS. The zeta potentials were all negative and decreased in magnitude in the most concentrated suspension (from −18 mV to −10 mV).

**Table 3:** Dynamic light scattering analysis of BaSO_4_ NM-220 suspensions.

Concentration (mg/ml dH_2_O)	d_H_ (nm)	PdI	ζ (mV)	Conductance (mS/cm)
0.67	222 ± 4	0.24 ± 0.01	−17.4 ± 1.9	0.02 ± 0.0
6.7	241 ± 5	0.37 ± 0.02	−18.6 ± 0.9	0.07 ± 0.01
33.3	388 ± 29	0.51 ± 0.08	−9.7 ± 0.6	0.15 ± 0.01

Data are mean ± SD, n = 3. d_H_, hydrodynamic diameter, PdI, polydispersity index, ζ, zeta potential.

**Figure 6:**
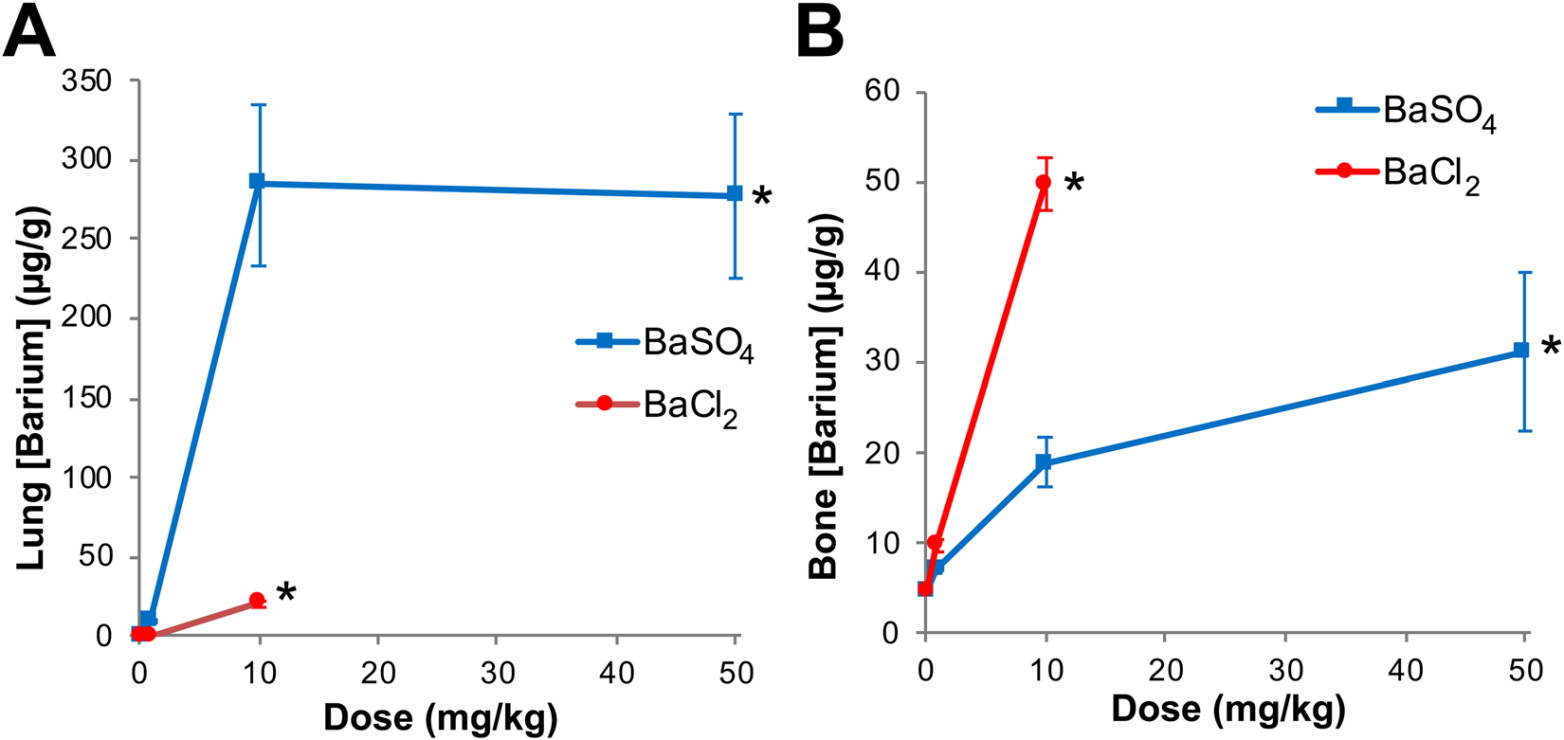
Study 2. Tissue concentrations of barium post-instillation of BaSO_4_ NPs or BaCl_2_ solution in rats. (**A**) Barium concentration in the lungs at 28 days post-instillation. There were significant dose-dependent increases in lung retention of barium post-instillation of BaSO_4_ nanoparticles and BaCl_2_ (*P < 0.001, ANOVA). Barium concentration in the lungs instilled with ionic barium (BaCl_2_) was lower than with BaSO_4_ NPs. (**B**) Barium concentration in bone (without bone marrow) at 28 days post-instillation. There were also dose-dependent increases in retained barium in bone (*P < 0.001, ANOVA). Significantly higher amounts of barium were retained in the bone of rats instilled with BaCl_2_. The slopes of bone barium concentrations were significantly different between BaCl_2_ and BaSO_4_ animals (P < 0.001, ANOVA). n = 4–5 rats per group. Note: Doses are for mass of BaSO_4_ NPs and BaCl_2_, not of barium. The % Ba in BaSO_4_ NPs and BaCl_2_ are 58.8 and 65.9, respectively.

To determine the contribution of NP dissolution in the biokinetics of barium post-instillation of BaSO_4_ NPs, we measured barium concentrations in the lungs and bones of rats instilled with increasing doses of BaSO_4_ NPs versus dissolved BaCl_2_ at 28 days post-instillation (Fig. 6, Table 4). First, there were significant increases in lung retention up to the 10 mg/kg dose. However, at the higher dose of 50 mg/kg, the amount of barium retained in the lungs did not increase, indicating faster clearance rates (Table 4, Fig. 6). Barium in ionic form (BaCl_2_) even at 10 mg/kg dose was mostly cleared by day 28. At 10 mg/kg dose, the lung barium concentration in the BaCl_2_ group was 20 µg/g compared to 285 µg/g from the BaSO_4_ NP group. In contrast with the lungs, significantly higher amounts of barium were retained in the bones of rats instilled with BaCl_2_ compared to rats administered with BaSO_4_ NPs (Fig. 6B). The bone and lymph node barium concentrations significantly increased with increasing dose of both BaSO_4_ NPs and BaCl_2_ (ANOVA, P < 0.001). The slopes of bone and lymph node barium concentrations between BaCl_2_ and BaSO_4_ animals were also significantly different (ANOVA, P < 0.001) (Table 4).

**Table 4:** Barium concentration and content in selected tissues of rats 28 days after intratracheal instillation of 1, 10 or 50 mg/kg BaSO_4_ nanoparticles and 1 or 10 mg/kg BaCl_2_.

Study 2		BaSO_4_			BaCl_2_		
Organ	Dose (mg/kg)	[Ba] µg/g	Organ Wt. (g)	Total Ba (µg)	[Ba] µg/g	Organ Wt. (g)	Total Ba (µg)
Lungs	0	0.01 ± 0.01^#^	1.10 ± 0.10	0.02 ± 0.02	0.01 ± 0.01^#^	1.10 ± 0.10	0.02 ± 0.02
	1	10.06 ± 0.61	1.17 ± 0.10	11.76 ± 0.57	0.21 ± 0.13*	1.30 ± 0.08	0.27 ± 0.17*
	10	284.6 ± 50.7	1.21 ± 0.09	344.4 ± 55.6	20.12 ± 1.87*	1.17 ± 0.13	23.4 ± 2.40*
	50	276.6 ± 51.5	1.30 ± 0.14	355.3 ± 17.0	N.D.		
Bone	0	4.71 ± 0.33^#^	16.8 ± 1.06	79.06 ± 6.85	4.71 ± 0.33^#^	16.8 ± 1.06	79.06 ± 6.85
	1	7.10 ± 0.27	18.3 ± 1.62	130.3 ± 12.9	9.74 ± 0.65	18.1 ± 0.76	176.3 ± 13.7*
	10	18.9 ± 2.75	18.1 ± 0.77	342.4 ± 48.8	49.78 ± 2.98*	17.2 ± 1.48	851.4 ± 31.1*
	50	31.2 ± 8.8	18.5 ± 1.87	562.5 ± 187.3	N.D.		
Bone Marrow	0	0.17 ± 0.19	10.7 ± 0.68	1.80 ± 1.85	0.17 ± 0.19	10.7 ± 0.68	1.80 ± 1.85
	1	0.07 ± 0.02	11.7 ± 1.03	0.81 ± 0.33	0.10 ± 0.02	11.6 ± 0.49	1.11 ± 0.22
	10	0.22± 0.05	11.6 ± 0.49	2.51 ± 0.61	0.24 ± 0.05	11.0 ± 0.95	2.63 ± 0.68
	50	0.24 ±0.04	11.8 ± 1.20	2.83 ± 0.76	N.D.		
Liver	0	0.006 ± 0.004	11.1 ± 1.54	0.07 ± 0.05	0.006 ± 0.004	11.1 ± 1.54	0.07 ± 0.05
	1	0.005 ± 0.002	12.2 ± 1.86	0.06 ± 0.02	0.007 ± 0.002	11.3 ± 1.02	0.08 ± 0.03
	10	0.007 ± 0.005	11.7 ± 0.89	0.08 ± 0.07	0.007 ± 0.001	10.9 ± 1.19	0.08 ± 0.02
	50	0.008 ± 0.001	12.8 ± 2.74	0.10 ± 0.02			
Lymph Nodes	0	0.07 ± 0.03^#^	N.D.		0.07 ± 0.03	N.D.	
	1	0.31 ± 0.51	N.D.		0.09 ± 0.03	N.D.	
	10	5.89 ± 5.73	N.D.		0.11 ± 0.03	N.D.	
	50	41.38 ± 10.2	N.D.		N.D.		

Data are mean SD, n = 5 per group.

Lymph nodes – tracheobronchial.

Total bone and bone marrow weights were estimated as 5% and 3.2% of body weight, respectively.

N.D., not determined, estimate of total lymph node mass in rats is not available. BaCl_2_ at 50 mg/kg was highly toxic.

*P < 0.001, Student’s T test, BaCl_2_ vs. BaSO_4_.

^#^P < 0.001, ANOVA. Dose-dependent increases.

Note: Doses are for mass of BaSO_4_ NPs and BCl_2_, not of barium. The % Ba in BaSO_4_ NPs and BaCl_2_ are 58.8 and 65.9, respectively

#### Transmission electron microscopic examination of lungs and other tissues in Study 2

**Figure 7:**
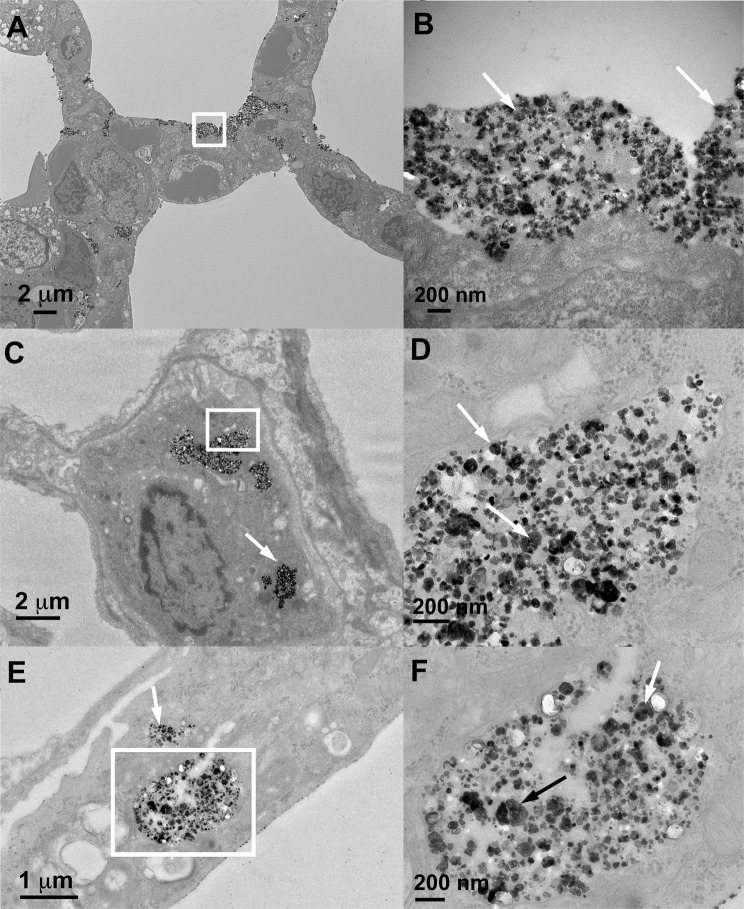
Study 2. Rat lung at 5 minutes (**A**,**B**) and 7 days (**C**–**F**) post-instillation of 10 mg/kg BaSO_4_ NPs. (**A**) BaSO_4_ NPs are shown lining the alveolar walls of rat lung immediately (5 minutes) post-instillation. The majority of BaSO_4_ NPs were located extracellularly. (**B**) Higher magnification of boxed area in panel A showing BaSO_4_ particles (arrows). (**C**) At 7 days post-instillation, alveolar macrophages with engulfed BaSO_4_ nanoparticles (arrows) within phagolysosomes. (**D**) Higher magnification of boxed area in panel C showing BaSO_4_ particles (arrows) in membrane-bound phagosomes. (**E**) Type II epithelial cell with internalized BaSO_4_ nanoparticles (arrows). (**F**) Higher magnification of boxed area in panel E showing BaSO_4_ particles (arrows) in membrane-bound vesicles in type 2 epithelial cell cytoplasm.

The cellular/tissue localization of retained BaSO_4_ NPs in the lungs of rats was examined by transmission electron microscopy. Figure 7 shows a rat lung immediately after and at 7 days post-IT instillation with 1 mg/kg BaSO_4_ nanoparticles. When we examined the rat lungs about 5 minutes after instillation, the majority of instilled particles were seen extracellularly in the alveoli interspersed within alveolar lining materials. Later at 7 days, the majority of BaSO_4_ NPs were seen within phagolysosomes in alveolar macrophages (Fig. 7C,D) and occasionally in type II epithelial cells (Fig. 7E,F). The BaSO_4_ NPs were densely packed within phagolysosomes and had a similar appearance to the original particle suspension delivered to the lungs (Fig. 5). In contrast, the BaSO_4_ particles inside the alveolar macrophages after 2 years of chronic inhalation were sparsely distributed and were interspersed with electron lucent materials (Fig. 2B). Individual particles were less electron dense and lacked the typical globular shape of the original NPs suggesting gradual dissolution.

#### Stimulated Raman Scattering microscopy of lungs and other tissues from IT-instilled rats

**Figure 8:**
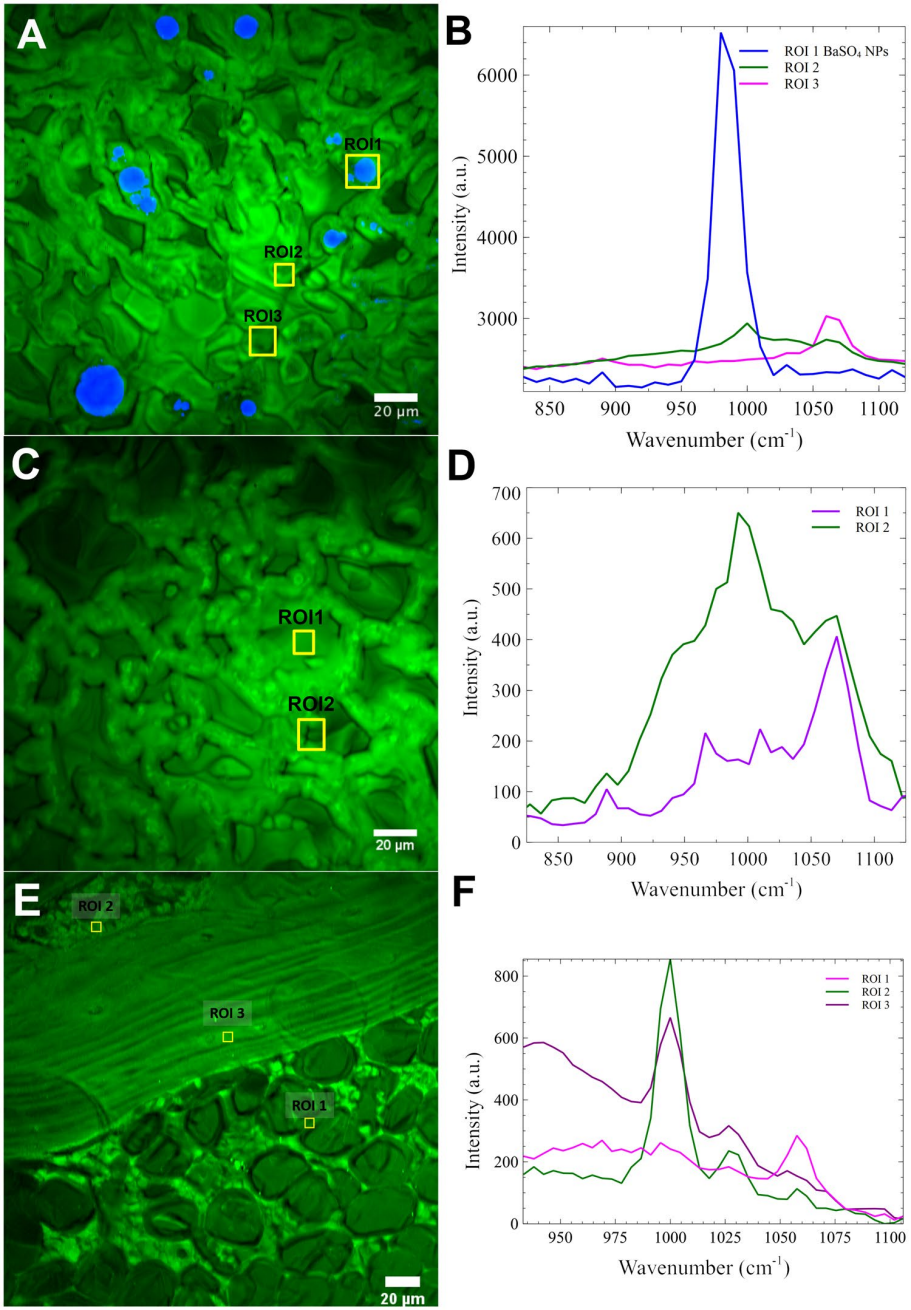
Study 2. Stimulated Raman spectroscopy microscopic examination of lungs and bone 28 days after IT instillation of 50 mg/kg BaSO_4_ NPs. (**A**) Areas with characteristic Raman spectrum of BaSO_4_ are shown in blue. (**B**) Raman spectra of three selected regions of interest (ROI) in panel A. Region of interest 1 (ROI 1) shows specific wavenumber for BaSO_4_. ROI 2 and ROI 3 did not show characteristic wavenumber for BaSO_4_. (**C**) Rat lung post- instillation of 10 mg/kg BaCl_2_. (**D**) No specific Raman spectrum of BaSO_4_ was detected in the lungs of rat instilled with BaCl_2_ solution. ROI 1 and ROI 2 are shown. (**E**) Rat bone at 28 days post- instillation of 50 mg/kg BaSO_4_ NPs. (**F**) No specific Raman spectrum of BaSO_4_ was detected. Regions of interest (ROI) 1, 2 and 3 are shown. Examination of the lungs, spleen, liver and kidneys in uninstilled control and in rats instilled with BaSO_4_ or BaCl_2_ did not show specific Raman spectrum of BaSO_4_ (data not shown).

To determine if BaSO_4_ NPs translocate to the bone as intact particles or as ionic barium, additional spectroscopic analysis of tissue samples from Study 2 were performed with SRS microscopy. This technique is based on identifying the characteristic Raman shifts of BaSO_4_ in unstained tissue sections. The lung, bone, liver, spleen and kidney of rats instilled with BaSO_4_ NPs or soluble BaCl_2_ were examined at 28 days post-instillation. Our results show that the signature Raman shift of BaSO_4_ was present in several foci in the lungs of rats instilled with BaSO_4_ NPs (Fig. 8A,B) but not with ionic BaCl_2_ (Fig. 8C,D). No signature Raman shift for BaSO_4_ was found in the control (uninstilled) lung (data not shown) nor in bone (Fig. 8E,F) of rats instilled with BaSO_4_ NPs or soluble BaCl_2_. No spectral signature of BaSO_4_ was detected in the liver, spleen and kidneys from control (uninstilled) and from BaCl_2_- and BaSO_4_-instilled rats (data not shown).

## Discussion

This study describes the fate of inhaled BaSO_4_ NPs and the barium they contain from their initial lung deposition until their retention in the lungs and other organs in a rat model. We have previously reported that lung clearance of barium from BaSO_4_ was far faster than cerium from CeO_2_ NPs (9.6 vs. 140 days), despite BaSO_4_ NPs having low dissolution rates in various simulant fluids^18,20^. The percentage of barium and cerium dissolved after 28-day incubation of BaSO_4_ and CeO_2_ NPs in phagolysosomal simulant fluid were 0.1% and <0.002% (lower than detection limit), respectively^18,20^. These data suggest that the dissolution rate of BaSO_4_ is different from CeO_2_ NPs in the lungs and that dissolution rates *in vivo* are different from those in cell-free simulant fluids. The present study sought to examine how much barium and in what forms are the barium retained in the lungs and selected organs (spleen, liver, bone, bone marrow and lymph nodes) after a 2-year inhalation exposure, and 4 weeks after IT instillation of BaSO_4_ NPs. To determine the contribution of particle dissolution on barium translocation from the lungs, we also examined barium retention in the same organs after IT instillation of ionic Ba delivered as BaCl_2_, a water-soluble salt of barium. *In toto*, our data suggest that the majority of barium from BaSO_4_ NPs translocates from the lungs only after particle dissolution and ion transport to primarily the bone. A small fraction of BaSO_4_ NPs translocate as intact particles to tracheobronchial but not to mesenteric lymph nodes.

After the final day of multiple inhalation exposure of rats in Study 1, the barium concentrations and estimated barium contents in the lungs, liver, lymph nodes, bone and bone marrow were measured. Based on these current data and on the previous reports in rats examined at 1, 28, and 90 days^18,28^, we found that barium continuously accumulated in the lungs up to 360 days. No change in barium content was seen between 360 days and 2 years, suggesting that deposition equaled clearance rates. The highest concentrations of barium in the exposed animals were measured in the lungs and lymph nodes (tracheobronchial and mediastinal) followed by the hard bone and bone marrow. The liver had the lowest concentration. The highest amounts of barium were found in the lungs as expected and in hard bone, consistent with both our previous^18^ and our current data from Study 2 in which rats were IT-instilled with the same batch of BaSO_4_ NPs.

Our data indicate that lung clearance of BaSO_4_ NPs becomes slower as lung burden increases, as reported in other studies^30,31^. Based on previous barium data from Study 1, the calculated lung clearance half-time from 90 days when the lung burden of barium was 1.6 mg/lung (1.24 mg/g lung weight) was approximately 56 days^28^. We have also shown that the lung burden after 28 days (0.84 mg/lung) decreased by 95% after 34 days^18^. Additionally, when deposited barium after IT instillation was 0.25 mg/lung (0.21 mg/g lung weight), the clearance half-time was shorter (9.5 days)^18^. Since no rats were continued after the end of the 2-year inhalation exposure, lung clearance when the retained barium was much higher could not be determined. The decreasing clearance with increasing lung barium burden increasing suggests that the clearance mechanisms, such as mucociliary clearance and NP dissolution in phagolysosomes, might have been impaired. However, we also observed that during the second year of continuous exposure, barium in the lungs did not increase further (Fig. 1A), indicating that the rate of deposition was similar to the rate of lung clearance. This observation suggests that the barium was translocated from the lungs through pathways and mechanisms not previously understood. We believe particle dissolution leads to translocation of barium ions across the air-blood barrier followed by uptake by bone and other organs. Interestingly, substantial amounts of BaSO_4_ particles translocated to the lymph nodes. Barium concentrations in the lymph nodes were found to be as high as in the lungs (Table 2).

The faster lung clearance of BaSO_4_ compared to other poorly soluble particles such as CeO_2_ and TiO_2_ suggests that BaSO_4_ solubility *in vivo* is substantially higher despite their similar poor solubility in cell-free simulant fluids. Indeed, dissolution rates *in vitro* simulated conditions are much slower than when particles are ingested by alveolar macrophages. It has been shown that dissolution rates of cobalt oxide particles were faster when taken up by human and canine alveolar macrophages *in vitro* than when these particles were incubated in cell-free media with simulated pulmonary conditions^32^.

Is it possible that BaSO_4_ NPs can more easily translocate as intact NPs through the air-blood barrier than CeO_2_ NPs? Do the high amounts of barium in the bone prove translocation of intact BaSO_4_ NPs from the lungs to the bone? To explore this possibility, we compared the biokinetics of barium post-instillation of BaSO_4_ NPs versus barium ions (BaCl_2_). We compared the barium concentrations in the lungs and bones of rats instilled with BaSO_4_ NPs versus barium ions (BaCl_2_ dissolved in water) at 28 days post-instillation. The amounts of barium remaining in the lungs increased with increasing mass dose of instilled BaSO_4_ NPs. However, the lung barium concentrations were the same with 10 and 50 mg/kg doses. Greater amounts of barium translocated to tracheobronchial lymph nodes when rats were dosed with 50 mg/kg (Table 4), similar to rats in Study 1. Lung injury, inflammation, and increased fluid exiting the lungs via the lymphatics when a higher dose of barium was delivered in a single bolus might have enhanced the clearance of barium from the lungs.

Ionic barium was cleared almost completely from the lungs by 28 days. This suggests that once BaSO_4_ NPs dissolve in the lungs, ionic barium can be translocated from the lungs to the circulation and other organs. Higher amounts of barium from BaCl_2_ compared with BaSO_4_ NPs were retained in the hard bone. These data indicate that the relatively higher bioavailability of barium after inhalation or instillation of BaSO_4_ NPs is due to NP dissolution and subsequent transport of ionic barium from the lungs into extrapulmonary sites especially the bone, bone marrow and lymph nodes. It is unlikely due to the movement of intact NPs across tight epithelial barriers.

Transmission electron microscopy was used to look for intact BaSO_4_ particles especially in tissues with high barium concentration. Whether IT-instilled or inhaled, the majority of retained BaSO_4_ NPs in the lungs were seen as particles within phagolysosomes in alveolar macrophages and less commonly in type II epithelial cells and neutrophils. We observed BaSO_4_ NPs within endosomes of phagocytic cells in tracheobronchial but not in the mesenteric lymph nodes despite their high barium concentrations. These particles could have reached these lymph nodes either as extracellular or more likely phagocytosed particles within macrophages from the lungs. Barium in mesenteric lymph nodes might have reached there as ionic barium from the tracheobronchial lymph nodes. The BaSO_4_ NPs in the lungs of IT-instilled rats were densely packed within phagolysosomes and had a similar appearance as that of the original particle suspension. However, after chronic inhalation, the particles were sparsely distributed, and the individual particles were less electron dense. These morphologic changes suggest gradual particle dissolution within the phagolysosomes over a period of months or more. Most of the BaSO_4_ particles were taken up by alveolar macrophages at 7 days post-instillation, similar to our previous observation in BAL cells from the lungs of rats at 24 hours after instillation of BaSO_4_ and CeO_2_ NPs^33^. These data indicate that BaSO_4_ NPs deposited in the lungs are readily phagocytosed by macrophages. No particles were seen in the bone marrow and liver, as well as in the lungs of air-exposed control and BaCl_2_-instilled rat lungs. All these findings suggest that intact BaSO_4_ NPs can translocate to anatomically close lymph nodes via the lymphatics. But few are likely to translocate into the circulation as free nanoparticles. Recent reports on other poorly soluble gold and CeO_2_ NPs also conclude that NP translocation across the air-blood barrier is a rare event^20,33–35^.

We also employed spectroscopic analysis of the same tissues using SRS microscopy to explore whether barium in multiple tissues was in the form of BaSO_4_ or not. This technique is based on identifying the characteristic Raman shifts of BaSO_4_ in unstained tissue sections. The lung, bone, liver, spleen and kidney of rats instilled with BaSO_4_ NPs or ionic BaCl_2_ were examined. The signature Raman shift of BaSO_4_ was present in several foci in the lungs of rats instilled with BaSO_4_ NPs but not with soluble BaCl_2_. No signature Raman shift for BaSO_4_ was found in the lungs, bone, liver, spleen or kidneys from uninstilled control, BaCl_2_- and BaSO_4_-instilled rats. Our findings from SRS further confirm that translocation of barium from BaSO_4_ NPs to extrapulmonary organs is not as intact particles.

## Conclusions

Our data indicate that barium translocates from the lungs after dissolution of BaSO_4_ NPs within alveolar macrophages. This conclusion is supported by comparing lung and bone barium concentrations in BaSO_4_ NP-instilled versus BaCl_2_-instilled rats. Furthermore, we found no evidence of specific BaSO_4_ Raman spectral characteristics in extrapulmonary organs. Thus, the abundant barium in bone and mesenteric lymph nodes must represent ionic barium’s incorporation into the bone matrix as it forms and do not represent uptake of intact NPs. Electron microscopic imaging show that some intact BaSO_4_ NPs are transported to lung-associated lymph nodes most likely via the lymphatic circulation, but not to distant (mesenteric) lymph nodes. Additionally, clearance of BaSO_4_ NPs from the lungs is faster than can be predicted from its very low dissolution in simulant fluids. Thus, our data also underscore the limitations of *in vitro* dissolution assays in predicting biokinetics *in vivo* and emphasizes that solubility in water or lung simulant fluids needs to be carefully interpreted.

The precise mechanisms of how barium is transported from the phagolysosomes to the blood, and to extrapulmonary organs deserves further study. Additional analyses using high resolution transmission electron microscopy to study bioprocessing of BaSO_4_ particles in the lungs, and the chemical/molecular forms of barium in the bone are underway.

## Methods

### Characterization of BaSO_4_ nanoparticles and BaCl_2_

The BaSO_4_ NPs (NM-220) used for these studies were a reference material prepared for the Nanomaterial Testing Sponsorship Program of the Organization for Economic Cooperation and Development (OECD). The characterization of the original batch distributed as NM-220 has been published^36^. Since Study 1 (2-year inhalation) requires large amounts, BaSO_4_ NPs were reproduced at a different production plant using the same synthesis protocol. This reproduced batch was characterized by the same methods. Barium chloride anhydrous beads (99.99% pure) were obtained from Sigma-Aldrich (St. Louis, MO) and were used to prepare a stock solution of BaCl_2_ at 50 mg/ml in distilled water.

### Experimental Design: Study 1 - Two-year inhalation exposure of rats to aerosol of barium sulfate nanoparticles

The protocols for the long-term inhalation studies were approved by the local authorizing agency in Landesuntersuchungsamt, Koblenz, Germany. The procedures for inhalation exposure, aerosol generation and the monitoring system have been described^18^. Female Wistar Han rats were obtained at 7 weeks of age from Charles River Laboratories (Sulzfeld, Germany). The animals were maintained in groups of up to 5 animals in a polysulfon cages (TECNIPLAST, Germany) with access to wooden gnawing blocks, GLP certified feed (Kliba laboratory diet, Provimi Kliba SA, Kaiseraugst, Basel, Switzerland) and water *ad libitum*. Rats were exposed to 50 mg/m³ BaSO_4_ aerosol or filtered air for 24 months (6 h/day, 5 consecutive days/week). The animals were exposed while in wire cages located in a stainless-steel whole-body inhalation chamber (V = 2.8 m^3^ or V = 1.4 m^3^). The aerosols entered the inhalation chambers with the supply air and were removed by an exhaust air system with 20 air changes per hour. When containing control animals, the chamber air pressure was above the room air pressure to ensure that no room air reaches the control animals. For the BaSO_4_-exposed rats, the chamber air pressure was negative to prevent contamination of the laboratory as a result of particle leakages from the inhalation chambers.

The majority of the cohort was used for histopathologic examination. One day after the last exposure, six rats (two air-exposed control and four BaSO_4_-exposed) were euthanized and tissue samples were collected and processed. Samples of lung, liver, lymph nodes, bone and bone marrow from the six rats were collected and frozen. These were analyzed for barium concentration using ICP-MS. Parts of the same organs were processed for electron microscopy as described below.

### Experimental Design: Study 2 - Intratracheal instillation of barium sulfate nanoparticles and barium chloride solution in rats

The protocols used in the instillation study were approved by the Harvard Medical Area Animal Care and Use Committee. Male Wistar Han rats (10 weeks old) were obtained from Charles River Laboratories (Wilmington, MA) and were housed in microisolator cages under controlled conditions of temperature, humidity, and light at the Harvard Center for Comparative Medicine. They were fed commercial chow (PicoLab Rodent Diet 5053, Framingham, MA) and reverse-osmosis purified water *ad libitum*. The animals were acclimatized in the facility for seven days before the start of experiments.

BaSO_4_ NPs were suspended in sterile distilled water at 0, 0.67, 6.7 or 33.3 mg/ml for intratracheal instillation (IT) at doses of 0, 1, 10 and 50 mg/kg body weight, respectively. BaCl_2_ was dissolved in sterile water at 0.67 and 6.7 mg/ml for 1 and 10 mg/kg doses, respectively. The volume dose was held constant at 1.5 ml/kg. Suspensions in tubes were sonicated with a Branson Sonifier S-450A (Branson Ultrasonics, Danbury, CT, USA) fitted with a cup sonicator at 242 J/ml, the critical dispersive energy shown to maximally disperse these particles in water^37^ while immersed in running cold water to minimize heating of the particles. The hydrodynamic diameter (d_H_), polydispersity index (PdI), conductance, and zeta potential (ζ) of each suspension were measured using a Zetasizer Nano-ZS (Malvern Instruments, Worcestershire, UK). Aliquots of each suspension were also examined under electron microscopy.

Each rat was weighed, and the volume dose of instilled particles was calculated. The NP suspension was loaded in a sterile syringe with attached blunt-tipped 18-gauge gavage needle. Each rat was then anesthetized with vaporized isoflurane (Piramal Healthcare, Bethlehem, PA), restrained on a slanted board, and held upright by their upper incisor teeth resting on a rubber band. The larynx was transilluminated with focused light for better visualization of the glottis during instillation. The tip of the needle was gently inserted into the trachea between the vocal cords, with the tip just above the tracheal bifurcation. The BaSO_4_ NP suspension or BaCl_2_ solution was gently delivered in one bolus.

Rats were humanely killed at 5 minutes (n = 2), 7 days (n = 2), and 28 days (n = 7) after dosing. Rats were anesthetized with vaporized isoflurane and euthanized by exsanguination via the abdominal aorta. The lungs, liver, tracheobronchial lymph nodes, bone marrow from the two femoral bones and femur (hard bone) were collected and placed in pre-weighed tubes. Barium concentration in each sample from 5 rats/group sacrificed at 28 days was measured using ICP-MS. Two rats euthanized at each of time points (7, 28 and 60 days) after instillation were used for morphologic examination of lungs, bone, spleen, liver and kidneys as described below.

### Tissue analysis of barium content using ICP-MS

Tissue samples from selected organs in rats from Study 1 and Study 2 were analyzed for barium concentrations. In Study 1, barium concentration in the lungs, lymph nodes (tracheobronchial and mesenteric), liver, bone and bone marrow of BaSO_4_ aerosol-exposed and air-exposed control rats were measured. In Study 2, barium concentration in the lungs, liver, tracheobronchial lymph nodes, bone marrow, and bone (without marrow) at 28 days post-instillation were measured. All samples were handled with special care to avoid contamination during processing. Samples were analyzed for barium by inductively coupled plasma mass spectrometry (ICP-MS) (Luna Nanotech, Ontario, ON, Canada). Tissue samples were digested with nitric acid for 24 h, and then further digested with hydrogen peroxide for 24 h at room temperature. Ultrapure water was used for final sample dilution.

### Electron microscopic examination of lungs and other tissues

In Study 1, lung and other tissue samples from two control and four BaSO_4_-exposed rats were collected and processed for electron microscopic examination. In Study 2, lungs from two rats per group were fixed in neutral buffered 10% formalin via the trachea at 30 cm water pressure. The bone, spleen, liver and kidneys were immersion-fixed in the same fixative. The fixed tissues were trimmed, paraffin embedded and sectioned. The sections were stained with hematoxylin and eosin. Lung, liver, spleen, kidney and decalcified bone sections were examined under light microscopy. Unstained paraffin-embedded tissue sections were examined using SRS microscopy.

Tissue samples from two rats per group from Study 2 euthanized at 5 minutes, and 7 and 28 days post-dosing were processed for electron microscopic examination of lung tissues. Each rat was anesthetized with vaporized isoflurane and were whole-body perfused with heparinized saline via the right ventricle, followed by 2.5% glutaraldehyde in HEPES buffer, pH 7. Fixed lungs were stored at 4 °C in the same fixative prior to processing and Epon embedding, sectioning and examination under electron microscopy.

### Stimulated Raman scattering microscopy of lungs and other tissues

SRS microscopy is a nonlinear optical imaging technique that employs two ultrashort laser pulses (pump and Stokes) to coherently excite a Raman vibration that has an energy corresponding to the energy difference between the pump and the Stokes pulses. The coherent excitation improves signal intensity with much higher efficiency than spontaneous Raman and also eliminates interference from background fluorescence^38^. Therefore, it has advantages over spontaneous Raman as a useful non-destructive and label-free chemical imaging technique^39^. The tissue samples were analyzed with an SRS microscope for the presence of spectral signature of BaSO_4_ which has a Raman peak at 980 cm^−1^. We imaged 5 random fields of view per slide to test for the presence of particulate BaSO_4_ in each paraffin-embedded unstained tissue section. Several regions of interest (ROI) were analyzed for Raman spectra to differentiate BaSO_4_-specific Raman peak from the surrounding tissue matrix. Additional details of SRS imaging protocols are provided in the Supplement.

### Statistical analyses

Differences in organ concentrations of barium of rats in Study 1 and 2 were analyzed using Student t tests and analysis of variance (ANOVA) followed by Ryan-Einot-Gabriel-Welsch Multiple Range *post hoc* tests. Statistical analyses were performed using SAS statistical analysis software (SAS Institute, Cary, NC).

### Ethical approval

Protocols for Study 1 were approved by the local authorizing agency in Landesuntersuchungsamt Koblenz, Germany. Animal experiments in Study 1 were performed following relevant OECD guidelines for Testing of Chemicals, Section 4: Health Effects, No. 453^38^. Animal experiments in Study 2 follow protocols in accordance with relevant guidelines and were approved by the Harvard Medical Area Animal Care and Use Committee (Boston, MA).

## Supplementary information

Molina et al. Online Supplement

## Data Availability

The datasets supporting the conclusion of this article are included within the article. There are 8 Figures and 4 Tables. All relevant raw data are freely available to researchers wishing to use them.
