# Supplementary material for: Fate of Barium Sulfate Nanoparticles Deposited in the Lungs of Rats
Source: Sci Rep. 2019 Jun 3;9:8163. doi: 10.1038/s41598-019-44551-2 (PMC6546789; doi:10.1038/s41598-019-44551-2)
Supplement: Supplementary file 1 — Molina et al. Online Supplement [file 41598_2019_44551_MOESM1_ESM.docx]

**­­­Online Supplement**

**Fate of Barium Sulfate Nanoparticles Deposited in the Lungs of Rats**

Ramon M. Molina ^a^ *, Nagarjun V. Konduru ^a^, Priscila M. Queiroz ^a^, Benjamin Figueroa ^b^, Dan Fu ^b^, Lan Ma-Hock ^c^, Sibylle Groeters ^c^, Dirk Schaudien ^d^ and Joseph D. Brain ^a^

^a^ Molecular and Integrative Physiological Sciences Program, Department of Environmental Health, Harvard T.H. Chan School of Public Health, 665 Huntington Avenue, Boston, MA 02115, USA.

^b^ Department of Chemistry, University of Washington, 36 Bagley Hall, Seattle, WA, 98195 USA.

^c^ BASF SE, Carl-Bosch-Straße 38, 67056 Ludwigshafen, Germany

^d^ Fraunhofer-Institute for Toxicology and Experimental Medicine ITEM

Nikolai-Fuchs-Str. 1, 30625 Hannover, Germany.

| **Table S1**. Physicochemical characterization of BaSO_4_ nanoparticles | | |
| --- | --- | --- |
| **Parameters** | **Study 1**  BaSO_4_  reproduced batch | **Study 2**  BaSO_4_  NM-220 batch |
| Crystallite size (XRD) | 22.5 nm | 36.0 nm |
| Crystalline phase (XRD) | Barite orthorhombic | Barite orthorhombic |
| Particle size distribution  TEM: primary particle diameter  State of Agglomeration  SEM: agglomerate diameter | 25 ± 10 nm  2,000 nm to 13,000 nm  spheres (SEM) | 25 ± 10 nm  2,800 nm to 15,000 nm  sphere (SEM) |
| Specific surface area  Hg-intrusion porosimetry  DIN 66133 | surface area: 38 m²/g  pore sizes:  30 nm, 200 nm, 5000 nm | surface area: 33 m²/g  pore sizes:  30 nm, 5000 nm |
| Surface chemistry  XPS | Ba 15  O 64  C 2  S 17  Na 2 | Ba 13  O 52  C 17  S 11  Cl 3  P 3  N 1 |
| Surface charge  Electrophoretic mobility with  pH titration | IEP at pH 3.5 ± 0.2  ζ-pot at pH 7: -2.4(µm/s)/V/cm)  ZP: -32 ± 2 mV | IEP at pH 3.3 ± 0.2  ζ-pot at pH 7: -2.2(µm/s)/V/cm)  ZP: -28 ± 2 mV |
| Photocatalytic activity  DIN methylene blue assay | 0.95 + 0.2 -0.95 x 10^-3^  (compatible with zero) | 1.1 + 0.2 -1.1 x 10^-3^  (compatible with zero) |
| Dispersability  AUC, ISO13318: D50/AAN* | 160 nm/6.4 (in water)  198 nm/5.4 (in DMEM/FCS) | 116 nm/4.6 (in water)  285 nm/11 (in DMEM/FCS) |
| Solubility (ICP-MS)  in water 1 d  in DMEM/FCS 1d  in PSF 28 d  in PBS 28 d  in FaSSIF 7 d  in 0.1N HCl 1 d |  | See Fig. 1 for SEM results  0.05 wt%  0.02 wt%  0.1 wt% (recrystallizes)  0.1 wt%  0.1 wt%  1.0 wt% |
| Purity  (combined assessment from XRD, ICP-MS, XPS, TGA data) | 95 % | 93.8 % |
| Impurities | Water and 1.8% organic additives (mass loss in TGA) | Water and 1.8% organic additives (mass loss in TGA) |
| XRD, x-ray diffraction; XPS, x-ray photoelectron spectroscopy; AUC, analytical ultracentrifugation; ICP-MS, inductively coupled plasma mass spectrometry; DMEM/FCS,Dulbecco’s modified Eagle medium/fetal calf serum; PSF,phagolysosomal fluid; PBS, phosphate buffered saline; FaSSIF, fasted state simulant intestinal fluid; TGA, thermogravimetry; AAN, average agglomeration number. Data are mean ± standard deviation | | |

**Additional Details of SRS imaging**.

A broadband femtosecond dual beam laser system (Insight DS+ from Spectra-Physics) was used for SRS imaging. The Insight DS+ provides dual output beams at an 80 MHz repetition rate; a tunable beam (pump) ranges from 680 to 1300 nm and a fixed beam (Stokes) at 1040 nm. The Stokes beam is modulated by an electro-optical modulator (EOM) at 20 MHz. Sample analyses were done by utilizing the spectral focusing approach (1), which mechanically delays one of the two beams to provide the scanning of the Raman excitation frequency when the pump and Stokes beams are appropriately chirped. The pump and Stokes beams are chirped by long high dispersion H-ZF52A glass rods to match the group velocity dispersion for increased spectral resolution (1). The SRS microscope provide a spectral resolution of ~25 cm^-1^. After spatial and temporal overlap of the two beams were satisfied, the beams were sent into a laboratory-built laser scanning microscope (NIKON Eclipse FN1). A Nikon microscope of 40X (CFI Apo Lambda S 40XWI) with 1.25 NA was used to focus the beams onto the samples. After filtering out the Stokes beam with a short pass filter FESH1000 (Thorlabs), the pump beam is detected by a 10 x 10 mm^2^ silicon photodiode (Hamamatsu) biased at 56 V and then amplified by a home-built transimpedance amplifier. SRS signal is detected with a Zurich Instruments lock-in amplifier (H2FLI). Each frame is 512 x 512 pixels, with a 4 μsec pixel dwell time. FOV was 200 x 200 μm^2^. To obtain a hyperspectral data set, the total acquisition time varied from 30 to 40 sec depending on the spectral range desired.

**References**

1. Fu D, Holtom G, Freudiger C, Zhang X, Xie XS. Hyperspectral imaging with stimulated Raman scattering by chirped femtosecond lasers. *J Phys Chem B*. 2013;117(16):4634-40.
